# Evaluation of the Effects of Different Surface Configurations on Stability of Miniscrews

**DOI:** 10.1155/2013/396091

**Published:** 2013-07-01

**Authors:** Tolga Topcuoglu, A. Altuğ Bicakci, M. Cihat Avunduk, Z. Deniz Sahin Inan

**Affiliations:** ^1^Department of Orthodontics, School of Dentistry, University of Gaziantep, Sehitkamil, 27310 Gaziantep, Turkey; ^2^Department of Orthodontics, School of Dentistry, University of Cumhuriyet, 58000 Sivas, Turkey; ^3^Department of Pathology, Meram Medical Faculty, University of Selcuk, 42030 Konya, Turkey; ^4^Department of Histology and Embryology, School of Medicine, 58000 Sivas, Turkey

## Abstract

*Introduction*. The aim of this study was to analyze the effects of screw design and force application on the stability of miniscrews, using RTT, SEM, and histomorphometric analyses. *Materials and Methods*. Eighty cylindrical, self-drilling, and Ti6Al4V alloy miniscrews (1,6 × 6 mm) were used. Four mini-screws were inserted in fibulas of each rabbit, and 115 *G* of force was immediately applied. Four miniscrews were inserted in the other fibula, on which no force was applied. Eight weeks after insertion, osseointegration between miniscrew and the surrounding bone was evaluated by the histomorphometric analyses, SEM, and RTT. Kruskal-Wallis and the paired *t*-tests were used for statistical analysis. *Results*. Values obtained from Group I were significantly higher than those of the other loaded groups (*P* < .05). There were no differences in RTT scores among Groups II, III, and IV. Similar findings were also observed for unloaded mini-screws. There was no significant difference between Groups I and I_C_, while the differences between loaded and unloaded controls for each miniscrew were statistically significant. *Conclusions*. Immediate loading of miniscrews does not impair screw stability. Also, the diameter of miniscrew and more frequent thread pitches have a positive effect on stability; however, length of miniscrews does not have a significant effect on the stability.

## 1. Introduction

Anchorage control, which is one of the most challenging problems in orthodontics, could be defined as resistance to unwanted tooth movement. Over the years, orthodontists have tried to solve the problem of unwanted movement by using devices, including various types of headgear, transpalatal arches, and other removable devices [1]. However, unwanted tooth movement, known as anchorage loss, is a major pitfall of these anchorage reinforcement methods. Within a decade, several kinds of noncompliance skeletal anchorage systems such as implants [2], onplants [3], mini plates [4], and mini- or microscrews [5–7] have gained in popularity among clinicians, as a means to obtain absolute orthodontic anchorage.

 Miniscrews, which were first introduced by Kanomi [5] to avoid unwanted tooth movement, can be easily placed into various locations in the alveolar bone due to their small dimensions. The insertion procedure does not involve extensive trauma, and they can bear load immediately after placement. Moreover, they are easy to remove and involve relatively lower treatment costs compared with implants and onplants [6, 8]. However, as a disadvantage, miniscrews can be removed easily with low removal torques compared with implants due to their small diameters and short lengths [6, 7].

 The main requirement of miniscrews is that they stay stable for the required period of time. In addition to histological evaluation, various biomechanical methods are available to evaluate the stability of miniscrews, such as measurement of resonance frequency [9, 10], and torque values [9, 11–13]. However, the removal torque test (RTT) is considered the most useful way to evaluate the mechanical relationship between the bone and the implant, in the clinical context [7, 14–17].

 In the literature, it has been reported that the success rate of miniscrews may vary from 87.5% to 100% [18–21] due to variations in screw design with regard to screw diameter, length, pitch shape, and screw material [6, 7, 22–24]. Retention of the implants also depends on the response of the supporting bone [8, 25]. Consequently, it is important to evaluate not only screw design, but also bone response, to clarify the success of miniscrews. Therefore, the aim of this study was to analyze the effects of screw design (length, diameter, and thread form) and force application on the stability of miniscrews, using RTT, scanning electron microscopy (SEM), and histomorphometric analyses.

## 2. Materials and Methods

The protocol for this study was approved by the Experimental Animal Committee of Cumhuriyet University. For the study, a total of 80 commercially available cylindrical, self-drilling, and Ti6Al4V alloy miniscrews of 1.6 mm diameter and 6 mm length, produced by 4 different companies (Abso-anchor, Dewimed, Dual-top, and Neo-anchor), were ordered from the dealers of the manufacturers. The inner and outer diameters, thread lengths, and interpitch distances of the miniscrews were measured in detail (AutoDesk AutoCAD 2007) (Table 1).

**Figure 1:**
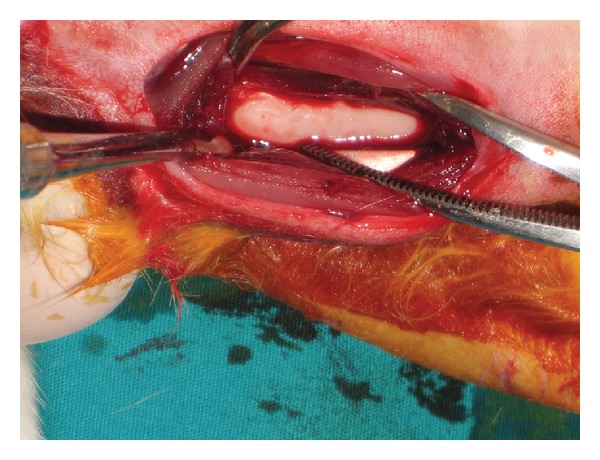
Image of the fibula after dissection.

**Figure 2:**
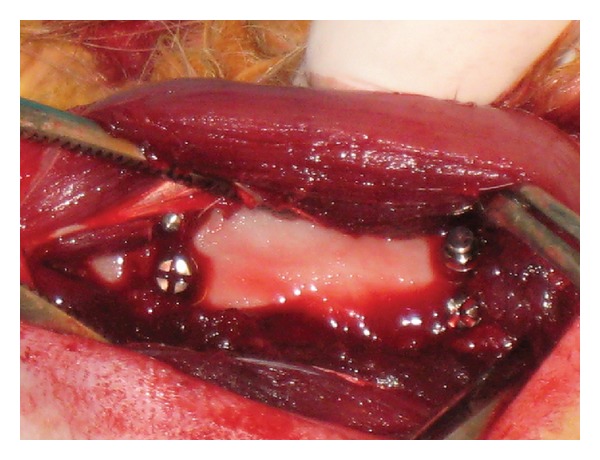
Four miniscrews placed in the fibula.

**Figure 3:**
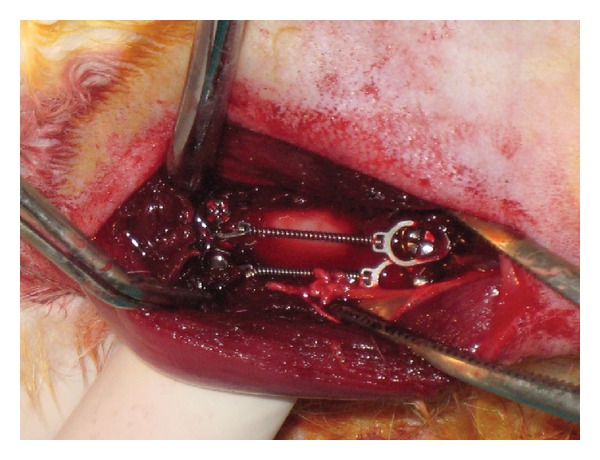
Four Miniscrews after 115 *G* of force applied with a nickel-titanium closed-coil spring.

In this study, ten 6-month-old male New Zealand white rabbits weighing 3.0–3.5 kg were used. All surgeries were performed under sterile conditions in a veterinary operating room. Rabbits were first anesthetized via intramuscular injection of ketamine hydrochloride (100 mg per kg) and xylazine (5 mg per kg), then the hair on the medial surfaces of the right and left fibulas was clipped, and the skin was cleansed with iodinate surgical soap. A 50 mm incision was made parallel to longitudinal axis of the fibula, and the periosteum was stripped (Figure 1). Miniscrews were placed into the first cortex of the fibula, and their longitudinal axes were adjusted parallel to each other and perpendicular to the external cortical fibula (without touching the second cortex) (Figure 2). Four miniscrews of different brands were placed in randomly selected fibulas of each rabbit, and 115 *G* of force was immediately applied using a nickel-titanium (Ni-Ti) closed-coil spring (TAD, GH Wire Company, Hanover, Germany; C2 size: medium, 15 mm) (Figure 3). Four miniscrews were placed in the other fibula, on which no force was applied; these comprised the control groups. All miniscrews were manually inserted with a hand-held screwdriver, by the same operator. The tissues were then closed with absorbable sutures, and carprofen (4 mg per kg) was given for 3 days after surgery to minimize infection risk; nevertheless, 1 rabbit died due to infection in the operated region.

Two months post surgery, the remaining 9 rabbits were sacrificed via an intravenous overdose of sodium pentothal. The fibulas were dissected, and 18 bone blocks containing 4 miniscrews were prepared, each with at least 2 mm of surrounding bone. The 8 miniscrews inserted into both fibulas of 1 rabbit were not removed and were carefully separated into 2 parts with a carbon disc under saline irrigation, in order to evaluate the bone-miniscrew interface using scanning electron microscopic (SEM) analysis (Leo 440 computer-controlled digital SEM).

**Figure 4:**
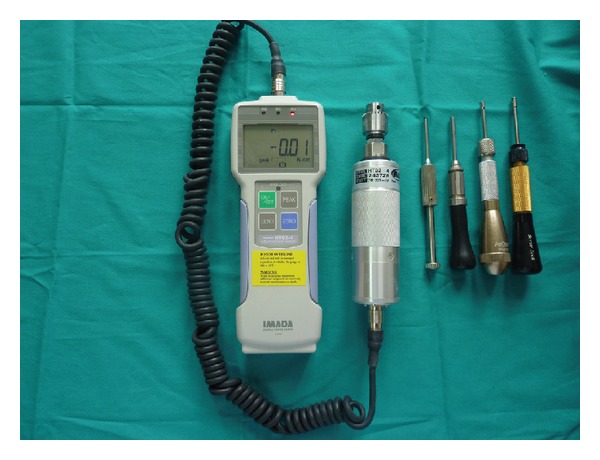
Portable digital torque gauge and screwdrivers.

In order to obtain removal torque values for the remaining 64 screws, a screw driver was placed into a digital portable torque gauge (HTG-2N, IMADA, Toyohashi, Japan) (Figure 4), and an incrementally increasing torque was applied until the miniscrew moved slightly within the adjacent bone. All measurements were performed by the same operator. The bone blocks containing miniscrews were then fixed in 10% buffered paraformaldehyde for 48 hours, followed by decalcification in ethylenediaminetetraacetic acid (EDTA) solution. After decalcification, the miniscrews were carefully removed. Tissue specimens were prepared in an Autotechnicon, embedded in paraffin, and sectioned with a microtome. The sections were stained with hematoxylin and eosin. Stained specimens were examined using a Nikon Eclipse E400 light microscope. For each specimen, the same area was photographed after staining, using a Nikon Coolpix 5000 photographic attachment. A photograph of each Nikon micrometer microscope slide (MBM11100, Japan) was also taken during the procedure. All photographs were then transferred to a PC and analyzed using the Clemex Vision Lite 3.5 Image Analysis program. Lengths were calculated by comparing the photograph of each specimen with the photograph of the Nikon micrometer microscope slide, which was taken under the same magnification. New bone formation in a designated 0.5 mm^2^ area was also evaluated, using the same image analysis program.

### 2.1. Statistical Analysis

Data obtained from histomorphometrical and biomechanical analyses were analyzed with the statistics software SPSS 16.0 (SPSS, Inc., Chicago, IL, USA). The difference was considered significant when *P* < .05. Differences between the groups with regard to removal torque values and new bone formation area were analyzed with Kruskal-Wallis and Tukey's post hoc test. The paired *t*-test was used to calculate intragroup differences in removal torque values.

## 3. Results

Five days after placement of miniscrews, 1 rabbit was excluded from the study due to infection around the operated region. The remaining 72 miniscrews that were placed in 9 rabbits remained stable, and no mobility was observed clinically during the 2-month experimental period.

There were no differences in RTT scores among Groups II, III, and IV; however, scores obtained from Group I were significantly higher than those of the other loaded groups (*P* < .05) (Table 2). Similar findings were also observed for unloaded miniscrews. While no difference was found among Groups II_C_, III_C_, and IV_C_, RTT scores obtained from Group I_C_ were significantly higher than those of the other controls (*P* < .05) (Table 2).

 Statistical analyses investigating the differences between loaded and unloaded controls for each miniscrew revealed that the differences between Groups II and II_C_, III and III_C_, and IV and IV_C_ were statistically significant, while there was no significant difference between Groups I and I_C_.

**Figure 5:**
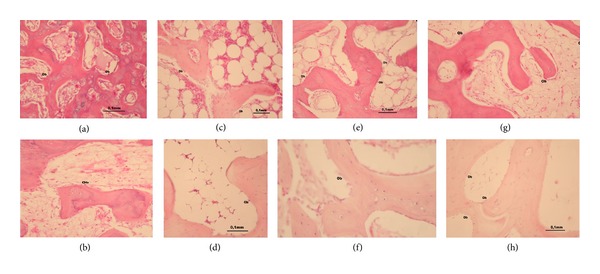
Images of hematoxylin-eosin-stained sections captured under light microscopy for loaded ((a) Group I; (c) Group II; (e) Group III; (g) Group IV) and unloaded ((b) Group I_C_; (d) Group II_C_; (f) Group III_C_; (h) Group IV_C_) groups.

 The results of histomorphological analysis showed that new bone formation per unit area (0.5 mm^2^) in the experimental groups was higher than that of their corresponding controls; however, the differences were not statistically significant (Table 3) (Figures 5(a)–5(h)).

**Figure 6:**
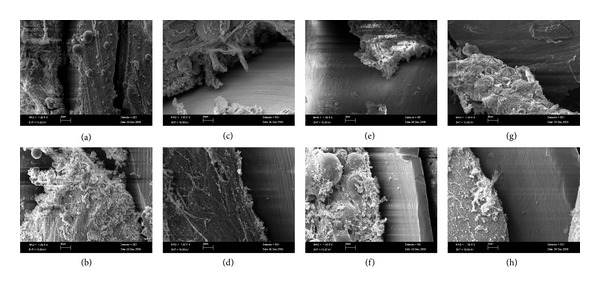
SEM images of loaded ((a) Group I; (c) Group II; (e) Group III; (g) Group IV) and unloaded ((b) Group I_C_; (d) Group II_C_; (f) Group III_C_; (h) Group IV_C_) groups, depicting the adaptation between the screw threads and the globular feature of bone-like tissue.

 SEM images showed that there were no morphological differences between the groups. Adaptation between miniscrew and the host bone, a globular entity of bone-like tissue, was seen in the screw threads of all loaded and unloaded groups (Figures 6(a)–6(h)).

## 4. Discussion

Achieving mechanical stability immediately after placement of miniscrews is crucial for obtaining primary stability. The amount of force applied on miniscrews is one of the important factors. Heavy forces may increase the mobility of miniscrews, preventing osseointegration, and may ultimately cause failure of miniscrews [26]. A wide range of applied forces (25–300 *G*) has been investigated in recent studies [7, 27–33]. In the present study, 115 *G* force was used, which was provided by Ni-Ti coil springs, in order to evaluate the stability of miniscrews under force application. It was demonstrated that 115 *G* force applied on the miniscrews modified the interfacial tissue between the screw and the bone, and this new tissue formation increased the success rates compared with the nonloaded screws. Remodeling of the bone around the loaded implants has been found to be more active than remodeling around unloaded implants [25, 34–38]. The increased bone repair around the loaded miniscrews could be attributed to the potential differentials and piezoelectric effects [39, 40] and to the functional adaptation mechanism of the bone in response to a changing mechanical environment [32, 41]. Furthermore, several clinical studies have shown that orthodontic force application has a positive effect on the stability of titanium miniscrews as well as positive effects on peri-implant bone [42–45]. Since increased bone healing is induced by biomechanical stimulation, in the present study it could be suggested that the higher removal torque values in the loaded groups compared with those of the unloaded controls were obtained due to the formation of new bone tissue.

 In a study conducted by Tseng et al., [46] a positive correlation was observed between the length and success rate of miniscrews. However, the results of other studies investigating the possible correlation between length and success rate revealed that the length of transitional implants was not related to the removal torque values [47]. Similarly, no significant increase in removal torque values with increasing screw length was found in the present study.

 Miyawaki et al. [48] showed that the success rates of miniscrews with diameters of 1.0, 1.5, and 2.3 mm were 0%, 83%, and 85%, respectively. Park et al. [26] achieved clinically acceptable success rates using miniscrews with diameters of 1.2, 1.5, and 2.3 mm. Although osseointegration has been reported around miniscrews with a diameter of 1 mm, clinically many failures have been reported [29, 33]. Based on these literature findings and the results of the present study, we can conclude that retention is distinctly correlated with the diameter of the miniscrews, because RTT values were positively correlated with screw diameter. However, the large increase in removal torque values in Group I could not be solely attributed to a difference in diameter of only 1 mm; the difference in diameters was also 1 mm between Groups II and III, but the difference in removal torque values between these groups was only 0.65 N/cm, while it was 1.58 N/cm between Groups I and II. The difference might be attributed to the thread design of the miniscrews in Group I. The “S” type thread design of the neck region may be a reason for the high removal torque values obtained from Group I. It is likely that this thread design increased intrabone screw cohesion and also provided better mechanical retention. Although the screws in Group IV had the lowest removal torque values, none of the miniscrews in this group showed any mobility during the study. 

It was possible that high removal torque values could have been proven to be problematic due to the associated difficulty in removing the miniscrews. However, no fractures were observed during the removal of any of the miniscrews in this study.

## 5. Conclusions

Immediate loading of miniscrews does not impair screw stability. On the contrary, loaded screws showed better stability than unloaded screws. While the length of miniscrews did not affect stability, a positive relationship was observed between screw diameter and stability, and more frequent thread pitch also had a positive effect on stability.

## Figures and Tables

**Table 1:** Measurements of miniscrews by AutoDesk AutoCAD 2007.

	Groups			
	I	II	III	IV
Inner diameter	1.2 mm	1.1 mm	1 mm	1 mm
Outer diameter	1.8 mm	1.6 mm	1.7 mm	1.5 mm
Thread length	4.7 mm	5.6 mm	5.5 mm	4.4 mm
Interpitch distance	0.694 mm	0.721 mm	0.693 mm	0.702 mm

**Table 2:** Prescription and removal torque values of miniscrews for all groups.

Group	No.	Subgroup (mm)	Removal torque values N/cm	Significance
Loaded	I	Neoanchor (1.8 × 4.7)	8.50 (2.41–10.05)	*
	II	Dewimed (1.6 × 5.6)	6.92 (2.76–8.48)	**
	III	Absoanchor (1.7 × 5.5)	6.27 (3.99–9.87)	**
	IV	Dual top (1.5 × 4.4)	5.78 (4.17–7.95)	**

Unloaded	I_C_	Neoanchor (1.8 × 4.7)	8.10 (4.94–9.35)	*
	II_C_	Dewimed (1.6 × 5.6)	4.63 (3.53–8.59)	**
	III_C_	Absoanchor (1.7 × 5.5)	4.59 (2.26–5.57)	**
	IV_C_	Dual top (1.5 × 4.4)	4.10 (2.59–5.53)	**

*P < .05 based on paired *t*-test.

**P < .05 based on post hoc Tukey's test.

**Table 3:** Representation of the osteoblast account in the per-unit area (0.5 mm^2^) of each group.

Groups (*n* = 5)	Osteoblast account
I	35 (26–43)
II	37 (23–45)
III	21 (15–38)
IV	26.50 (16–31)
I_C_	33 (18–46)
II_C_	24 (18–45)
III_C_	24 (15–42)
IV_C_	17.50 (16–29)
